# Sexual orientation in transgender adults in the United States

**DOI:** 10.1186/s12889-023-16654-z

**Published:** 2023-09-15

**Authors:** Sari L. Reisner, Soon Kyu Choi, Jody L. Herman, Walter Bockting, Evan A. Krueger, Ilan H. Meyer

**Affiliations:** 1 Department of Medicine, Harvard Medical School, 221 Longwood Ave, 5th Floor, Boston, MA 02115 USA; 2 Department of Epidemiology, Harvard T.H. Chan School of Public Health, Boston, MA USA; 3 Division of Endocrinology, Diabetes and Hypertension, Brigham and Women’s Hospital, Boston, MA USA; 4 The Fenway Institute, Fenway Health, Boston, MA USA; 5 Department of Research and Evaluation, Kaiser Permanente Southern California, Pasadena, CA USA; 6 The Williams Institute, School of Law, University of California Los Angeles, Los Angeles, CA USA; 7 Program for the Study of LGBTQ+ Health, Columbia University Irving Medical Center, New York, NY USA; 8 School of Social Work, Tulane University, New Orleans, LA USA

**Keywords:** Transgender, Sexual orientation, Sexual and gender minority, Health, Measures

## Abstract

**Background:**

Sexual orientation refers to a person’s enduring emotional, romantic, or sexual attractions to other people. Sexual orientation measures do not typically consider desires for, or sexual behavior with, transgender people. We describe measures inclusive of transgender people and characterize sexual orientation identity, behavior, and attraction in a representative sample of the U.S. transgender population.

**Methods:**

Between April 2016-December 2018, a U.S. national probability sample of transgender (*n* = 274) and cisgender (*n* = 1,162) adults were invited to complete a self-administered web or mailed paper survey. We assessed sexual identity with updated response options inclusive of recent identity terms (e.g., queer), and revised sexual behavior and attraction measures that included transgender people. Multiple response options were allowed for sexual behavior and attraction. Weighted descriptive statistics and sexual orientation differences by gender identity groups were estimated using age-adjusted comparisons.

**Results:**

Compared to the cisgender population, the transgender population was more likely to identify as a sexual minority and have heterogeneity in sexual orientation, behavior, and attraction. In the transgender population, the most frequently endorsed sexual orientation identities were “bisexual” (18.9%), “queer” (18.1%), and “straight” (17.6%). Sexually active transgender respondents reported diverse partners in the prior 5 years: 52.6% cisgender women (CW), 42.7% cisgender men (CM), 16.9% transgender women (TW), and 19.5% transgender men (TM); 27.7% did not have sex in the past 5 years. Overall, 73.6% were “somewhat”/ “very” attracted to CW, 58.3% CM, 56.8% TW, 52.4% TM, 59.9% genderqueer/nonbinary-females-at-birth, 51.9% genderqueer/nonbinary-males-at-birth. Sexual orientation identity, behavior, and attraction significantly differed by gender identity for TW, TM, and nonbinary participants (all *p* < 0.05).

**Conclusions:**

Inclusive measures of sexual orientation captured diverse sexual identities, partner genders, and desires. Future research is needed to cognitively test and validate these measures, especially with cisgender respondents, and to assess the relation of sexual orientation and health for transgender people.

## Background

Transgender is an umbrella term that describes people whose gender identity differs from the sex assigned to them at birth [1]. Gender identity is a person’s self-concept as female, male, both, or neither. For example, a person who identifies their gender as a man and was assigned a female sex at birth is transgender. Transgender people have diverse gender identities [2]. Some identify with a traditional male–female binary conceptualization of gender (e.g., transgender woman, transgender man). Others identify their gender in a nonbinary way (NB; e.g., genderqueer, genderfluid, bigender, agender), and may identify as neither male nor female, both male and female, or genderless. Best practices for U.S. national gender identity data collection were released in 2014 by The Williams Institute [3], and more recently in 2022 by the National Academies of Sciences, Engineering, and Medicine [4].

Transgender people can be of any sexual orientation [4]. Sexual orientation is multidimensional and refers to a person’s enduring emotional, romantic, or sexual attractions to other people [5]. Conceptually, sexual orientation comprises three dimensions: identity (how a person identifies their sexual orientation), behavior (the sex or gender of a person’s sexual partners), and attractions (the sex or gender of individuals that a person feels attracted to). Best practices for asking questions about sexual orientation in U.S. national population surveys were consolidated in 2009 by The Williams Institute [5], and articulated again in 2022 by the National Academies of Sciences, Engineering, and Medicine [4].

Sexual orientation categories traditionally include identity as straight or heterosexual, gay or lesbian, or bisexual; behavior as sexually active with men, women, or both; and attractions as a range of attractions of only, mostly, or equally attracted to males and/or females [6]. Sexual orientation terminology continues to evolve. For example, *queer* is historically a pejorative slur that has since been reclaimed by many sexual and gender minority people. Further, a major limitation of previous research has been that the most frequently-used sexual orientation measures did not account for transgender people [7–9], including people’s sexual behavior or attractions to transgender people and transgender people’s own sexual behavior or attractions. Further, studies have shown that transgender people endorse diverse sexual identities, including selecting “something else” when this is offered as a response option [10, 11]. Additionally, when given as a response option, “queer” is the most commonly reported sexual orientation identity in many transgender non-probability sample studies [2, 12]. Yet *queer*, is not included as a response option in U.S. population survey measures [5, 13].

Measures of sexual behavior and sexual attraction need to account for transgender people as sexually desirable or as sexual partners. Traditionally, sexual behavior and attraction measures in the U.S. have asked respondents to check a single response option [5, 6]. For sexual behavior, this is described as sexual behavior with women and men, exclusively or not; for sexual attraction, a range of response options are anchored in attraction to males or females [5, 8]. However, to capture sexual diversity, sexual behavior and attraction measures need to go beyond the traditional woman-man and female/male binary options. Moreover, measures that offer the opportunity for respondents to select multiple response options particularly for sexual behavior which may capture more accurate data on the gender of their sexual partners.

The aim of the study is to characterize sexual orientation identity, behavior, and attraction by gender identity in a probability sample representative of the U.S. population. This study describes updated measures of sexual orientation (identity, behavior, attraction) inclusive of transgender people. We compare the prevalence of sexual orientation identity, behavior, and attraction between transgender and cisgender participants. We also compare prevalence of sexual orientation identity, behavior, and attraction across three distinct subgroups of the transgender population (i.e., transgender women, transgender men, and nonbinary participants).

## Methods

### Participants and procedures

We use data from TransPop (www.transpop.org), a U.S. national probability sample of transgender adults conducted in collaboration with Gallup [14]. We used multi-stage sampling to assemble a probability sample of the U.S. adult population between April 2016 and December 2018. We used two recruitment methods: first, using random digit dialing to call landlines and cell phones and second, following industry trends, address-based sampling. The study also sampled a national probability sample of cisgender participants collected for comparison to the transgender sample. The recruitment period for cisgender participants was much shorter because there are so many more cisgender individuals than transgender individuals, but it was spread over time to reduce bias due to history (i.e., events unrelated to the study occurring at the time of recruitment).

We first identified transgender people among the general U.S. population sample via a screen questionnaire. Individuals identifying as transgender and/or having a gender identity different than the sex assigned to them at birth, along with meeting other eligibility criteria, were eligible and were invited to complete a self-administered web-based survey or mailed paper–pencil survey. Transgender people who identified as nonbinary were also eligible. Respondents who did not identify as transgender or whose current gender identity was the same as their sex assigned at birth were invited to participate in the cisgender survey by completing the self-administered questionnaire. The other eligibility criteria included: age 18 years or older, 6^th^ grade education or higher, and ability to complete the questionnaire in English. The final sample is 1,436 people, including 274 transgender participants and 1,162 cisgender participants. TransPop methods are described in detail elsewhere, including study design, sample size determination, implementation, and weighting procedures [14].

Participants included in the current analysis completed the demographic questions and the new measures of sexual orientation identity, behavior, and attraction designed for the study. Missingness across variables was < 5%.

## Measures

### Sexual orientation identity, behavior, and attraction

Table 1 presents the sexual orientation measures recommended in 2009 by SMART (column I) [5] and our updated sexual orientation (identity, behavior, and attraction) and sexual functioning measures (column II). The measure development and adaptation process were deductive and theoretically derived [15]. We drew from foundational concepts in sexual orientation to elaborate a working definition of the constructs we sought to measure (i.e., sexual identity, behavior, attractions). We then conducted a literature review to identify and evaluate existing sexual orientation measures. The research team, comprised of investigators with multidisciplinary expertise in sexual and gender minority populations and survey research methods, convened and collectively adapted the sexual orientation measures for transgender inclusion. The updated measures were then reviewed by subject matter experts, many of whom were transgender, including members of the TransPop Scientific Advisory Board and the Generations Study team [16]. Survey items were then pre-tested [17] to ensure items were well-worded, understood by the target population (both transgender and cisgender people), collected the information they were designed to measure, in order to identify potential sources of measurement error.

**Table 1:** Measures of sexual orientation identity, behavior, and attraction

I - SMART 2009	II - TransPoP 2017					
**Identity:**	**Identity:**					
Do you consider yourself to be:	Q 32. Which of the following best describes your current sexual orientation?					
□ Heterosexual or straight;	□ Straight/ Heterosexual					
□ Gay or Lesbian; or	□ Lesbian					
□ Bisexual?	□ Gay					
	□ Bisexual					
	□ Queer					
	□ Same-gender loving					
	□ Other: [write-in]					
**Behavior:**	**Behavior:**					
In the past (time period e.g. year) who have you had sex with?	Q 33. In the last 5 years, who did you have sex with? By sex we mean any activity you personally define as sexual activity. Please mark all that apply					
□ Men only,	□ Women, Non-Transgender					
□ Women only,	□ Men, Non-Transgender					
□ Both men and women,	□ Transgender Women/ Male-to-Female (MTF)					
□ I have not had sex	□ Transgender Men/ Female-to-Male (FTM)					
	□ I have not had sex with anyone in the last 5 years					
**Attraction:**	**Attraction:**					
People are different in their sexual attraction to other people. Which best describes your feelings? Are you:	Q 34. Please indicate how sexually attracted you are to the following types of people.					
		Not at all	Not very	Somewhat	Very	Not sure
□ Only attracted to females?	Women, Non-Transgender	□	□	□	□	□
□ Mostly attracted to females?	Men, Non-Transgender	□	□	□	□	□
□ Equally attracted to females and males?	Transgender Women/ Male-to-Female (MTF)	□	□	□	□	□
□ Mostly attracted to males?	Transgender Men/ Female-to-Male (FTM)	□	□	□	□	□
□ Only attracted to males?	Females at birth, Genderqueer	□	□	□	□	□
□ Not sure?	Males at birth, Genderqueer	□	□	□	□	□

#### Sexual orientation identity

For sexual orientation identity, response options were added to be inclusive of sexual orientation identities commonly endorsed by transgender people. Participants were asked to check the single response option that best described their current identity. Response options were straight/ heterosexual, lesbian, gay, bisexual, queer, same-gender loving, and other. We also used a variable that dichotomized these responses to sexual minority versus non-sexual minority by combining all responses that indicated a sexual minority identity versus the straight/heterosexual response option.

#### Sexual behavior

We revised the sexual behavior measure to be inclusive of sex with transgender people. Sexual behavior was assessed by asking people who they had sex with in the last 5 years. *Sex* was defined as anything that the respondent chose to describe as sexual activity. Response options were women, non-transgender; men, non-transgender; transgender women, transgender men, and “I have not had sex with anyone in the last 5 years.” Multiple response options were allowed for sexual behavior.

#### Sexual attraction

The measure of sexual attraction was updated to be inclusive of transgender people. Sexual attractions were assessed by asking how sexually attracted participants were to the following types of people with responses: women, non-transgender; men, non-transgender; transgender women; transgender men; females at birth, genderqueer (AFAB nonbinary); males at birth, genderqueer (AMAB nonbinary). Multiple response options were allowed. Response options for sexual attraction were on a Likert scale ranging from “not at all” to “very attracted,” and included a “not sure” response option. For analyses these were dichotomized as “somewhat attracted” “very attracted” and “not very attracted”, “not at all attracted”.

### Gender identity

Gender identity was assessed using response options to three questions asking about the respondents’ sex assigned at birth and current gender identity. Respondents were asked, “On your original birth certificate, was your sex assigned as female or male?” with answer options “Female” or “Male”. Respondents were then asked, “Do you currently describe yourself as a man, woman, or transgender?” with “Man”, “Woman” and “Transgender” as answer options. If the respondent identified as “Transgender” they were asked “Are you…” with answer options, “Trans Woman (Male-to-female)”, “Trans Man (Female-to-male)”, or “Non-binary/Genderqueer”.

### Other demographic characteristics

We asked about respondents’ age in years, race, ethnicity, and educational attainment.

## Data analysis

In analyses we compared the transgender to the cisgender population. If the respondent identified their current gender identity as transgender or their current gender identity was different from their sex assigned at birth (e.g., identified as man but was assigned female at birth) they were categorized as transgender. If the respondent’s current gender identity was the same as their assigned sex as birth, they were categorized as cisgender. We then disaggregated transgender respondents into three groups: transgender men, transgender women, and nonbinary respondents for analyses. Due to small sample size, we were not able to disaggregate the nonbinary group by assigned sex at birth.

Participants included in analyses were those who completed the demographic questions and the updated measures of sexual orientation (identity, behavior, and attraction) designed for the study.

We first compared the transgender to the cisgender population, then assessed gender identity subgroups (transgender men, transgender women, nonbinary individuals) within the transgender population. Data are weighted to adjust to response bias on gender, age, education, census region, and race and ethnicity. Weighted descriptive statistics were calculated; t-tests assessed statistical differences in age (continuous specification) by gender identity. χ^2^ tests were used to estimate differences in other demographics, sexual orientation, behavior, and attraction by gender identity. Age-adjusted models were fit to adjust estimates for the differing distribution of age in transgender and cisgender samples. Analysis of transgender respondents are weighted to represent the transgender adult population in the U.S. and analysis of cisgender respondents is representative of cisgender adults in the U.S. Analyses were conducted in SAS 9.1.

## Results

### Sample characteristics

Transgender respondents (*n* = 274) had a of mean age of 34.2 (SD = 14.8) and cisgender respondents (*n* = 1162) had a mean age of 48.5 (SD = 17.0) (Table 1). Mean age for transgender men was 30.4 (SD = 13.6), transgender women 40.4 (SD = 15.2), and nonbinary individuals 30.4 (SD = 12.6) (Table 3). Most transgender respondents were under the age of 40 (69.1%), whereas most cisgender respondents were 40 + (67.2%). A large proportion of transgender men (52.4%) and nonbinary individuals (45.2%) were between the ages 18–24.

More cisgender people (72.3%) said they were White than transgender people (56.5%) and conversely, more transgender people (43.5%) said they were people of color than cisgender people (27.7%). The distribution of racial and ethnic groups across transgender men, transgender women, and nonbinary individuals were similar, with 54%-59% of people identifying as White. A higher proportion of cisgender people had some or more college education than transgender people. Among transgender people, the distribution of educational attainment across transgender men, transgender women, and nonbinary individuals was similar.

### Sexual identity

**Fig. 1:**
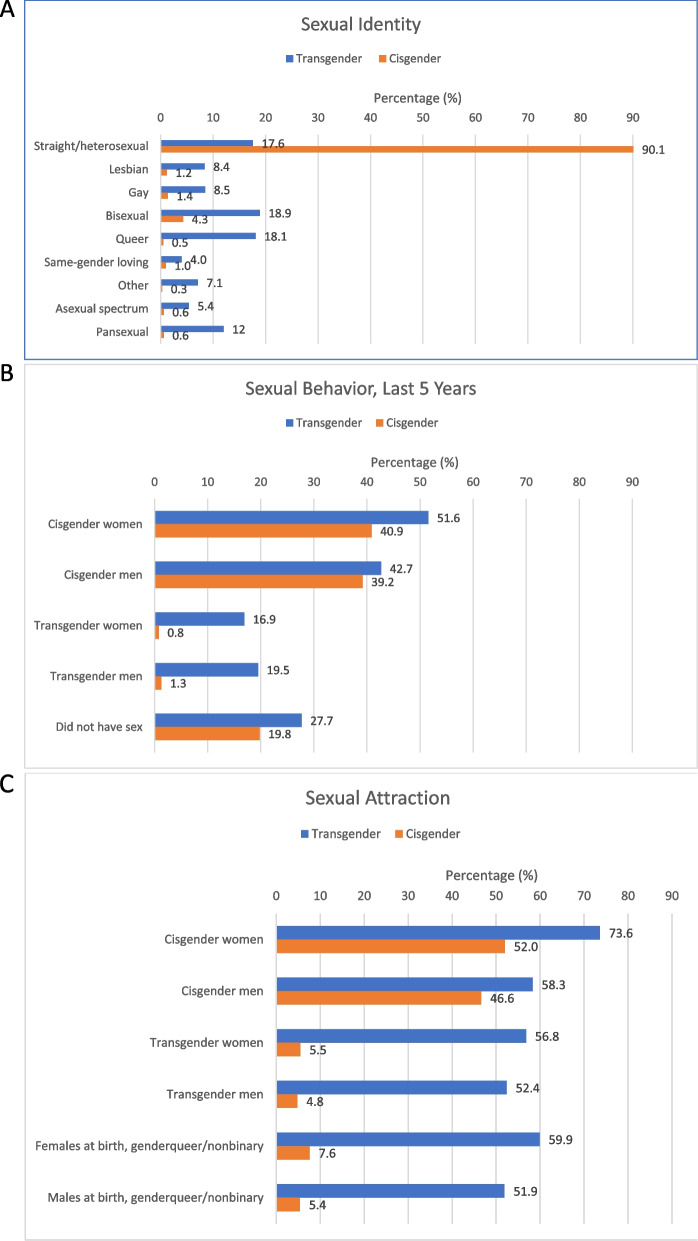
Sexual Identity (**A**), Behavior (**B**), and Attraction (**C**) Among Transgender and Cisgender Respondents. *Note:* The horizontal axis range for panels **A**, **B**, and is 0% to 95% in Fig. 1

Among transgender respondents, 82.4% had a sexual minority identity. The most common identities (Table 2, Fig. 1A) were bisexual (18.9%) and queer (18.1%), followed by straight/heterosexual (17.6%). Among cisgender people, 90.1% identified as straight/heterosexual, 9.9% had a sexual minority identity, with bisexual the most common (4.3%).

**Table 2:** Transgender and Cisgender Population Demographics and Sexual Orientation (Identity, Behavior, Attraction), Weighted % (*n* = 1,436)

	**Transgender (*n* = 274)**	**Cisgender (*n* = 1,162)**	**Test statistic**	***p*-value**	**Age-adjusted**	***p*-value**
**Mean age in years (SD)**	34.2 (14.8)	48.5 (17.0)	-14.00	< 0.001^a^	–	–
**Age group in years**			18.26	< 0.001	–	–
18–24	36.8	11.4				
25–29	12.2	7.4				
30–39	20.1	14.0				
40–49	12.6	17.7				
50 +	18.4	49.5				
**Race/ ethnicity**			5.51	< 0.001	1.73	0.141
White	56.5	72.3				
Black	9.5	11.1				
Latinx	15.7	9.2				
Multiracial	10.4	4.5				
Other	7.9	2.9				
**People of color**			12.97	< 0.001	3.90	0.048
Yes	43.5	27.7				
No, white	56.5	72.3				
**Education**			3.79	0.010	2.87	0.035
High school diploma or less	44.0	31.9				
Some college	31.2	31.6				
College graduate	14.3	19.9				
Post graduate work or degree	10.5	16.6				
**Sexual identity**			25.39	< 0.001^b^	20.36	< 0.001
Straight/ heterosexual	17.6	90.1				
Lesbian	8.4	1.2				
Gay	8.5	1.4				
Bisexual	18.9	4.3				
Queer	18.1	0.5				
Same-gender loving	4.0	1.0				
Other	7.1	0.3				
Asexual spectrum	5.4	0.6				
Pansexual	12.0	0.6				
**Sexual minority**			214.51	< 0.001	163.59	< 0.001
Any sexual minority identity	82.4	9.9				
Heterosexual/ straight	17.6	90.1				
**Sexual behavior in past 5 years**						
Did not have sex	27.7	19.8	4.29	0.039	2.65	0.104
**Sexual partner gender in past 5 years**						
Cisgender women	51.6	40.9	5.73	0.017	4.08	0.044
Cisgender men	42.7	39.2	0.65	0.421	0.08	0.777
Transgender women	16.9	0.8	25.15	< 0.001	22.19	< 0.001
Transgender men	19.5	1.3	27.72	< 0.001	12.58	0.0004
**Sexual attraction (somewhat or very attracted)**						
Cisgender women	73.6	52.0	21.48	< 0.001	7.21	0.007
Cisgender men	58.3	46.6	6.76	0.009	4.11	0.043
Transgender women	56.8	5.5	135.50	< 0.001	86.63	< 0.001
Transgender men	52.4	4.8	114.19	< 0.001	67.71	< 0.001
Females at birth, genderqueer/ nonbinary	59.9	7.6	146.48	< 0.001	92.96	< 0.001
Males at birth, genderqueer/ nonbinary	51.9	5.4	127.60	< 0.001	77.71	< 0.001

^a.^ T-test was conducted to assess statistical difference of means

^b.^ Test statistic is unstable due to small sample size (*n* < 10) in categories that are greyed out

Trans man 30.9%, trans woman 30.8%, trans GNB 31.3%

**Fig. 2:**
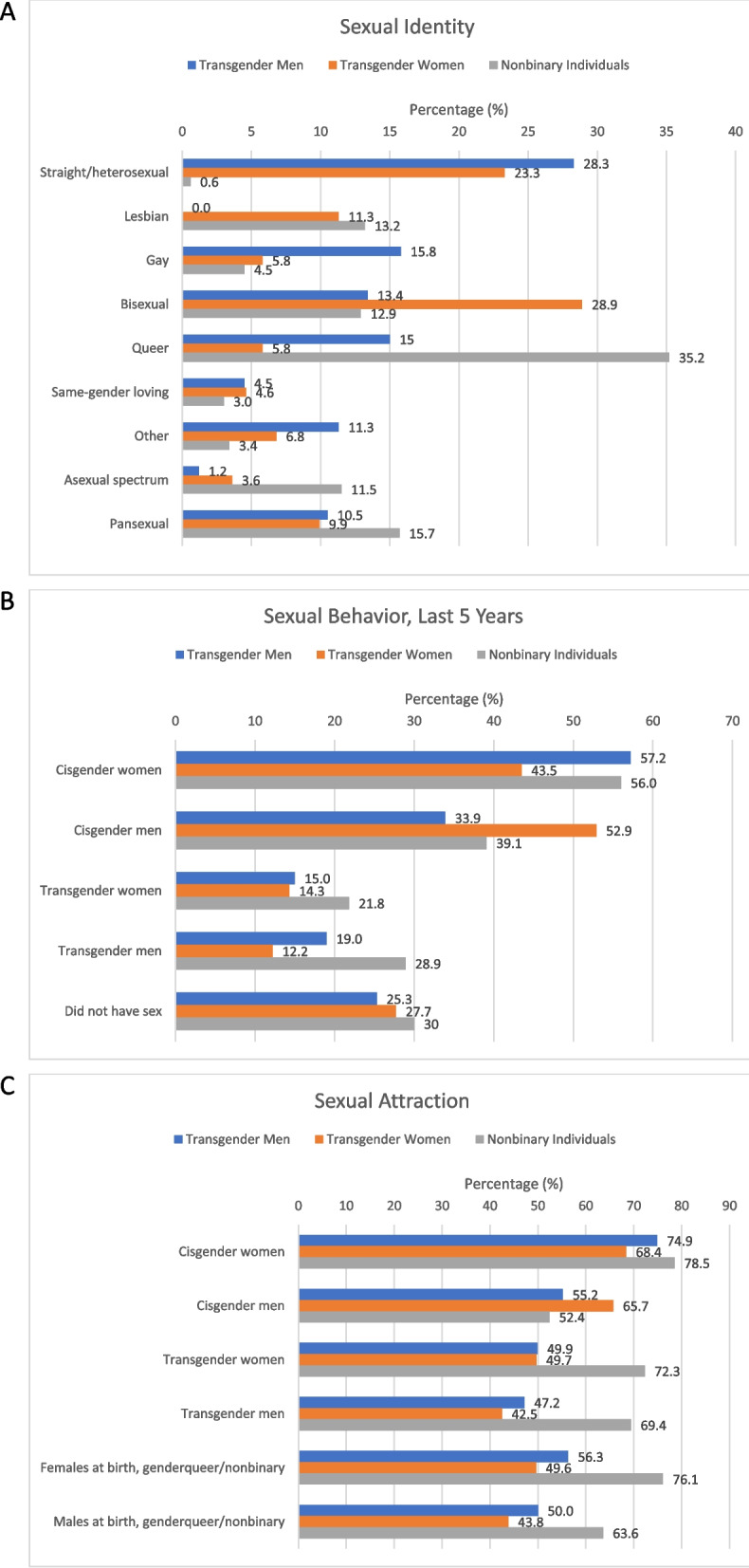
Sexual Identity (**A**), Behavior (**B**), and Attraction (**C**) Among Transgender Respondents: Transgender Men, Transgender Women, and Nonbinary Individuals. *Note:* The horizontal axes differ for Panel **A** (0% to 40%), **B** (0% to 70%), and **C** (0% to 90%) in Fig. 2

Among transgender men, 28.3% identified as straight/heterosexual, 15.8% identified as gay and 15.0% as queer. Among transgender women, 28.9% identified as bisexual, 23.3% as straight/heterosexual and 11.3% as lesbian. Among nonbinary individuals, 35.2% identified as queer, 15.7% identified as pansexual, 12.9% identified as “bisexual”, and 11.5% as asexual. Due to small sample sizes, we did not report estimates for any groups with *n* < 10. Overall, 71.7% of transgender men, 76.7% of transgender women, and 99.4% of nonbinary individuals identified as a sexual minority and the differences across the three groups was statistically significant (Table 3, Fig. 2A).

**Table 3:** Demographics and Sexual Orientation (Identity, Behavior, Attraction) in the Transgender Population by Gender Identity (*n* = 274)

	**Transgender Men (*N* = 78)**	**Transgender Women (*N* = 120)**	**Nonbinary Individuals (*N* = 76)**	**Test statistic**	***p*-value**	**Age-adjusted**	***p*-value**
**Mean age in years (SD)**	30.4 (13.6)	40.4 (15.2)	30.4 (12.6)	-16.39	< 0.001 ^a^	–	–
**Age group in years**				3.14	0.002	–	–
18–24	52.4	17.0	45.2				
25–29	5.5	11.1	20.1				
30–39	15.5	28.0	15.0				
40–49	15.1	13.1	9.6				
50 +	11.5	30.8	10.1				
**Race/ ethnicity**				0.81	0.594	1.03	0.415
White	55.4	59.3	54.2				
Black	12.6	7.7	8.5				
Latinx	16.6	10.1	21.7				
Multiracial	5.8	13.2	11.5				
Other	9.6	9.7	4.1				
**People of color**				0.150	0.859	0.21	0.812
Yes	44.6	40.7	45.8				
No, white	55.4	59.3	54.2				
**Education**				0.830	0.549	1.19	0.310
High school diploma or less	50.8	40.7	41.3				
Some college	30.7	36.3	25.2				
College graduate	10.3	13.8	19.0				
Post graduate work or degree	8.2	9.2	14.5				
**Sexual identity**				138.76	< 0.001^b^	70.49	< 0.001
Straight/ heterosexual	28.3	23.3	0.6				
Lesbian	0	11.3	13.2				
Gay	15.8	5.8	4.5				
Bisexual	13.4	28.9	12.9				
Queer	15.0	5.8	35.2				
Same-gender loving	4.5	4.6	3.0				
Other	11.3	6.8	3.4				
Asexual spectrum	1.2	3.6	11.5				
Pansexual	10.5	9.9	15.7				
**Sexual minority**				14.89	< 0.001	14.76	< 0.001
Any sexual minority identity	71.7	76.7	99.4				
Heterosexual/ straight	28.3	23.3	0.5				
**Sexual behavior in past 5 years**							
Did not have sex	25.3	27.7	30.0	0.11	0.896	0.14	0.870
**Sexual partner gender in past 5 years**							
Cisgender women	57.2	43.5	56.0	1.26	0.285	1.11	0.333
Cisgender men	33.9	52.9	39.1	2.28	0.104	3.67	0.027
Transgender women	15.0	14.3	21.8	0.58	0.562	0.48	0.617
Transgender men	19.0	12.2	28.9	2.13	0.120	1.21	0.300
**Sexual attraction (somewhat or very attracted)**							
Cisgender women	74.9	68.4	78.5	0.69	0.501	0.37	0.690
Cisgender men	55.2	65.7	52.4	1.19	0.306	3.63	0.028
Transgender women	49.9	49.7	72.3	3.30	0.038	2.61	0.075
Transgender men	47.2	42.5	69.4	3.93	0.021	2.33	0.099
Females at birth, genderqueer/ nonbinary	56.3	49.6	76.1	3.73	0.025	2.32	0.101
Males at birth, genderqueer/ nonbinary	50.0	43.8	63.6	2.05	0.131	0.95	0.389

^a.^ ANOVA was conducted to assess statistical difference of means

^b.^ Test statistic is unstable due to small sample size (*n* < 10) in categories that are greyed out

### Sexual behaviors

Respondents reported diverse sexual partners in the 5 years prior to survey: Among transgender respondents, 51.6% reported sex with cisgender women, 42.7% with cisgender men, 16.9% with transgender women, and 19.5% with transgender men. Approximately one-quarter (27.7%) of respondents did not have sex in the 5-year period. Among cisgender respondents, 40.9% reported sex with cisgender women, 39.2% with cisgender men, 0.8% with transgender women, and 1.3% with transgender men. 19.8% did not have sex in the 5-year period. Overall, among respondents sexually active in the last 5 years, 52.5% of transgender and 94.8% of cisgender people endorsed having exclusively one sexual partner gender (i.e., monosexual), 21.4% of transgender and 3.7% of cisgender people indicated two sexual partner genders, and 26.1% of transgender and 1.5% of cisgender people reported ≥ 3 or more sexual partner genders (Fig. 1B).

For transgender men, 57.2% reported sex with cisgender women, 33.9% with cisgender men, 15.0% with transgender women, and 19.0% with transgender men in the past 5 years. In TW, 43.45% reported sex with cisgender women, 52.9% with cisgender men, 14.3% with transgender women, and 12.2% with transgender men. Among nonbinary individuals, 56.0% reported sex with cisgender women, 39.1% with cisgender men, 21.8% with transgender women, and 28.9% with transgender men. Approximately one-quarter (25.3%) of transgender men, 27.7% of transgender women, and 30.0% of nonbinary individuals did not have sex in the 5-year period. The differences across the three groups were not statistically significant. Overall, among respondents sexually active in the last 5 years, 60% of transgender men, 58.5% of transgender women, and 37.0% of nonbinary individuals endorsed having exclusively one sexual partner gender (i.e., monosexual); 19.3% of transgender men, 16.4% of transgender women, and 29.8% of nonbinary individuals indicated two sexual partner genders; and 20.6% of transgender men, 25.1% of transgender women, and 33.2% of nonbinary individuals reported ≥ 3 or more sexual partner genders (Fig. 2B).

### Sexual attraction

Among transgender respondents, 73.6% were “somewhat” or “very” attracted to cisgender women, 58.3% to cisgender men, 56.8% to transgender women, 52.4% to transgender women, 59.9% to AFAB nonbinary, and 51.9% AMAB nonbinary. Among cisgender respondents, 52.0% were attracted to cisgender women, 46.6% to cisgender men, 5.5% to transgender women, 4.8% to transgender men, 7.6% to AFAB nonbinary, and 5.4% to AMAB nonbinary. All differences between cisgender and transgender respondents are statistically significant at *p* < 0.05. Respondents endorsed multiple attractions, with 63.9% of transgender and 5.9% of cisgender people reporting attractions to ≥ 3 partner genders (Fig. 1C).

Among transgender men, 74.9% were “somewhat” or “very” attracted to cisgender women, 55.2% to cisgender men, 49.9% to transgender women, 47.2% to transgender men, 56.3% to AFAB nonbinary, and 50.0% to AMAB nonbinary. Among transgender women, 68.4% were attracted to cisgender women, 65.7% to cisgender men, 49.7% to transgender women, 42.5% to transgender men, 49.6% to AFAB nonbinary, and 43.8% to AMAB nonbinary. Among nonbinary individuals, 78.5% reported being sexually attracted to cisgender women, 52.4% to cisgender men, 72.3% to transgender women, 69.4% to transgender men, 76.1% to AFAB nonbinary, and 63.6% to AMAB nonbinary. Respondents endorsed multiple attractions, with 58.2% of transgender men, 57.1% of transgender women, and 77.8% of nonbinary individuals reporting attractions to ≥ 3 partner genders (Fig. 2C).

## Discussion

This transgender population study utilized inclusive measures of sexual orientation that captured diversity of sexual identities, partners, and desires among transgender people. The measures of sexual orientation designed for this study highlight the range of sexual and gender diversity in the transgender population, providing an opportunity to report broader categories beyond those historically queried. Relative to the cisgender population, a higher proportion of the transgender population identifies as a sexual minority. The most reported sexual identities were bisexual and queer, highlighting the importance of having included response options beyond gay, lesbian, and bisexual, which are traditionally included in survey research. Further, the distribution of sexual partner genders and sexual attractions was more heterogenous for the transgender than cisgender population. Relative to cisgender people, a higher proportion of transgender people endorsed cisgender women, transgender women, and transgender men as sexual partners in the last 5 years. Transgender people also had a more even spread of sexual attractions across gender groups, relative to cisgender people, who primarily reported attractions to cisgender women and men.

Transgender respondents endorsed multiple sexual behaviors and attractions, demonstrating the need for multiple response options, and response options beyond the woman-man and female/male binary for transgender respondents. Our findings show that when multiple response options are allowed, many transgender participants endorse more than two partner genders in sexual behaviors and sexual attractions. Traditionally, sexual behavior and attraction measures have asked respondents to check a single response option [5]. For sexual behavior, this is described as sexual behavior with women and men, exclusively or not. For sexual attraction, response options are anchored in attraction to males and females. Our measures were updated with the understanding that sexual and gender diversity exists. To capture this diversity, we adapted the sexual identity response options to reflect the current sociocultural milieu and sexual orientation identities being commonly used by transgender people. Likewise, sexual behaviors and attractions response options are beyond the woman-man and female/male gender/sex binary. We assess behaviors with and attractions to transgender and nonbinary people by assigned birth sex, as well as to cisgender people by gender/sex (female, male). Challenges and solutions have been highlighted in collecting sexual orientation and gender identity data [18]. Our study builds upon and enhances these in sexual orientation measures inclusive of transgender people.

We recommend sexual identities, behaviors, and sexual attractions—using the plural form, rather than singular (e.g., identity, behavior, attraction)—be used by researchers. This terminology will further highlight the inclusivity and diversity of identities, behaviors, and attractions in the transgender population. The updated measures are inclusive of plurisexualism wherein people may self-identify as queer or pansexual, and may engage in sexual behaviors or experience sexual attractions to multiple genders and/or to those outside of the gender binary [19]. The measures are also inclusive of those who self-identify as asexual and who may have a low or absent desire for sexual activity [20]. The measures can be used to describe the distribution and diversity of sexual orientation identities, behaviors, and attractions inclusive of transgender people in future research. We offer updated measures for each of the three dimensions of sexual orientation given that the most relevant dimension(s) of sexual orientation for health research may be specific to the particular outcomes under study.

Findings can be considered alongside several limitations. Sample size limitations precluded disaggregation of the nonbinary group by assigned sex at birth. We examined sexual attractions as a binary variable for this same reason. Given that a high proportion of younger age cohorts may self-report as sexual minority [21] or endorse sexual behavior or attractions beyond the binary, we present age-adjusted comparisons. Yet, the study is largely descriptive without adjustment for other confounders. Future research is needed that explores age differences, including stratification of sexual identity, behaviors, and attractions by age groups. We note that we used several terms in the updated measures which are already less common. Language and terminology evolve quickly. We used the term “non-transgender” because at the time of the survey the term “cisgender” was not as commonly used as today, but that could be used (and is recommended for use), as a synonym today. Several response options, for example “transgender women/male-to-female (MTF)” and “transgender men/female-to-male (FTM)”, may also warrant revision. A limitation of this study is that the sexual behavior question had only four response options, while the sexual attraction measure had six response options and offered response options to indicate attractions to nonbinary AFAB and AMAB individuals. We suggest future research field the sexual behavior item and allow for six response options to be inclusive of nonbinary people. This was not a study to assess the validity or reliability of the measures. We sought to describe and present the measures and characterize sexual orientation, behavior, and attractions in a probability sample of the U.S. population by gender identity. Cognitive testing and a validity/reliability study represent a logical next step in this line of research.

## Conclusions

To our knowledge this is the first probability sample to examine "queer" as a sexual orientation identity category in the transgender population. Future research is needed to cognitively test and validate updated measures of sexual orientation, especially with cisgender respondents and in different languages (e.g., Spanish), and to assess the relation of sexual orientation and health outcomes for transgender people. Using updated measures of sexual orientation inclusive of transgender people, this U.S. nationally representative sample highlights the diversity of sexual orientation identities, behaviors, and attractions in the transgender population. This study contributes to the evidence-base demonstrating sexual orientation diversity in the transgender population and offers updated tools for transgender population health research.

## Data Availability

The dataset supporting the conclusions of the article is available from ICPSR at the University of Michigan: https://www.icpsr.umich.edu/web/DSDR/studies/37938.

## Abbreviations

TW: Transgender women
TM: Transgender men
NB: Nonbinary
GQ: Genderqueer
